# Is Asthma Remission a New Achievable Goal? A Review of the Current Drug‐Related Evidence and Predictors

**DOI:** 10.1111/all.70388

**Published:** 2026-05-14

**Authors:** Fatma Esra Gunaydin, Giulia Marcassa, Bianca Olivieri, Gianenrico Senna, Marco Caminati

**Affiliations:** ^1^ Allergy and Immunology Unit Frimley Park Hospital Surrey UK; ^2^ Department of Medicine University of Verona Verona Italy; ^3^ Allergy and Asthma Unit University Hospital of Verona Verona Italy

**Keywords:** airway remodeling, asthma, biologics, remission, severe asthma

## Abstract

Advances in asthma therapies have shown that clinical remission may be an achievable therapeutic goal for patients with asthma. This review discusses the current definitions of remission in asthma, the predictors of remission, airway inflammation/epithelial damage in asthma remission, airway remodeling, paradigms in defining remission and super‐responders, remission criteria in severe asthma studies with biologics, and future perspectives. The terminology of asthma remission basically describes a high level of disease control, which includes the absence of symptoms and exacerbations, avoiding the use of oral corticosteroids and stability of lung function with/without continuation of treatment. Predictors associated with spontaneous remission include younger age at asthma onset, milder symptoms at diagnosis, male sex, and higher baseline lung function. Biologic therapies have shown considerable success in achieving remission in asthma; however, predictors of response to these therapies remain heterogeneous and are not consistently defined across studies.

## 1 Introduction

In the last decades, the approach to asthma management has undergone a paradigm shift, evolving from the concept of asthma control to that of asthma remission. Traditionally, asthma management focused on symptom control through a stepwise increase in therapy aimed at minimizing exacerbations and maintaining daily functioning. However, with the advent of biologics, the goal of asthma treatment has expanded to include the potential for achieving remission [1].

The concept of asthma remission, unlike control, implies a long‐term absence of symptoms and exacerbations, and while traditionally restricted to patients who became symptom‐free either spontaneously (“spontaneous remission”) or after completing treatment (“remission off treatment”), it can also be achieved with ongoing therapy (“on‐therapy remission”), such as optimized inhaled therapies, including triple inhalation therapy, and/or biologics [2, 3, 4].

The term “remission” refers to the suppression or absence of biological disease activity. In asthma, however, the assessment of underlying airway inflammation remains challenging in routine clinical practice, as direct measurement requires invasive procedures and currently available biomarkers only partially reflect airway inflammatory activity. For this reason, the concept of “clinical remission” has been adopted, primarily based on symptom control, exacerbation absence, and functional stability. As a consequence, clinical remission may overlap with related concepts such as “super‐response”, which primarily reflects an exceptional treatment effect, or “complete response”, a more stringent state that requires objective evidence of inflammatory and functional normalization.

Defining remission in asthma remains a challenge [5, 6]. Current definitions vary and may include clinical, functional, and biomarker‐related criteria and continue to evolve with the increasing use of biologic therapies, particularly as some patients achieve symptom‐free status while still receiving treatment for severe asthma [7].

This review summarizes current definitions of asthma remission, examines the role of novel therapies in achieving remission, and the ongoing efforts to establish a universal, clinically applicable definition of remission in asthma. It is based on post hoc analyses of randomized controlled trials, as well as real‐world observational studies, evaluating approved biologic therapies for asthma. Studies were selected based on their clinical relevance and scientific credibility, with a particular focus on outcomes related to remission. Eligible studies reported standardized measures of asthma control (e.g., Asthma Control Test, ACT, or Asthma Control Questionnaire, ACQ), lung function parameters, exacerbation outcomes, oral corticosteroid (OCS) use and/or clinical remission rates over defined follow‐up periods.

## 2 Asthma Remission Definitions

**FIGURE 1:**
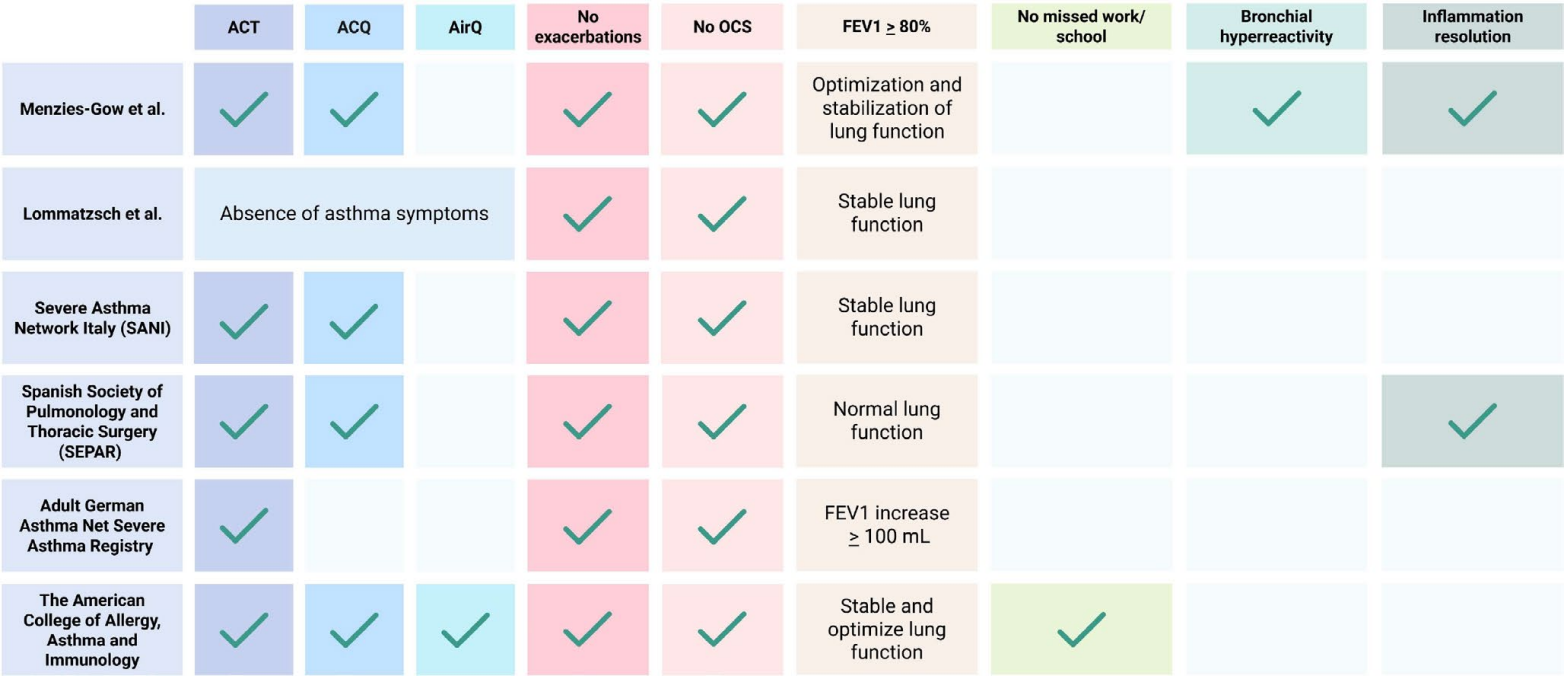
Comparative summary of the key domains included in the main definitions of asthma remission. ACQ, Asthma Control Questionnaire; ACT, Asthma Control Test; AirQ, Asthma Impairment and Risk Questionnaire; FEV1, Forced Expiratory Volume in 1 s; OCS, Oral corticosteroids.

Asthma remission definitions vary across studies and guidelines, with differences in the required duration of remission, treatment status, and the criteria used to assess it. Most definitions share core clinical elements, e.g., the absence of exacerbations, good symptom control, and no use of oral corticosteroids, typically sustained for at least 12 months, but differ in how these are defined and combined (Figure 1). Earlier definitions, such as that proposed by Carpaij et al. [8], required a stringent approach, including complete symptom resolution, discontinuation of asthma medications, and no lung function impairment or bronchial hyperresponsiveness.

In 2020, Menzies‐Gow et al. [9] proposed a more pragmatic definition of asthma remission using a modified Delphi approach, addressing the limitations of previous definitions, which primarily described spontaneous cessation of asthma symptoms unrelated to treatment. These earlier definitions, while informative, lacked applicability as practical treatment goals [10, 11, 12]. This framework aligns with remission concepts in other chronic inflammatory diseases and acknowledges that, while remission off treatment remains the ideal goal, remission on treatment, excluding systemic corticosteroids, represents a meaningful and achievable outcome. *Clinical remission* involves at least 12 months of symptom absence, stable lung function, patient‐provider agreement on the remission status, and no systemic corticosteroid use. *Complete remission*, on the other hand, adds objective criteria such as the absence of bronchial hyperresponsiveness and resolution of asthma‐related inflammation, measured by reduced blood or sputum eosinophils, fractional exhaled nitric oxide (FeNO), or other measures [9].

Similarly, Lommatzsch et al. [1] proposed a definition *of clinical disease remission* in asthma, closely linked to the use of disease‐modifying anti‐asthmatic drugs (DMAADs), focusing on symptom absence, no exacerbations, stable lung function, and no need for systemic corticosteroids.

Other definitions differ in the thresholds used for symptom control and the inclusion of additional domains. The American College of Allergy, Asthma and Immunology, together with the American Academy of Allergy, Asthma and Immunology, defined *clinical remission on treatment* based on multiple components, such as the absence of exacerbations and absenteeism from work or school, and good symptom control (ACT score > 20, AirQ score < 2, ACQ score < 0.75) [13]. While this definition may be useful in capturing deep clinical stability, its applicability may be limited by the strictness of its criteria, making it difficult to achieve in many real‐world populations treated with biologics or optimized inhaled therapy.

The German registry definition relies on ACT ≥ 20 combined with absence of exacerbations and oral corticosteroid use [14], whereas the Spanish Society of Pulmonology and Thoracic Surgery (SEPAR) consensus requires both clinical control and normal lung function, and introduces a more stringent definition of complete remission incorporating inflammatory markers (FeNO levels below 40 ppb and sputum eosinophils below 2%) [15]. The Japanese consensus similarly integrates symptom control (ACT ≥ 23 or ACQ ≤ 0.75 in adults), exacerbation absence, and optimization of lung function, while allowing for either normalization or stabilization in the presence of fixed airway limitation [16].

Additional definitions have expanded the concept of remission beyond clinical criteria. Rial et al. [17] propose the concept of what they termed “inflammatory remission”, which may be described as “biologic remission”, based on normalization of airway or serum biomarkers of inflammation, such as eosinophils, IgE, periostin, or FeNO, yet variability in obstruction and hyperresponsiveness can persist. However, whether and to what extent normalization of these biological parameters (either spontaneously or under treatment) predicts sustained long‐term remission remains unclear.

A Delphi consensus conducted by the Severe Asthma Network Italy (SANI) [18] proposed a distinction between *complete clinical remission* and *partial clinical remission*, requiring the absence of oral corticosteroid use even for partial remission. The study also touched upon *inflammatory remission*, focusing on biomarkers like eosinophils and FeNO, but this concept did not reach full consensus.

The National Jewish Health panel also incorporates biomarker‐based definitions of remission while allowing continued background therapy [19].

Asthma remission definitions remain heterogeneous, and while most focus on clinical criteria, some expert groups have proposed the additional concept of “complete remission” as a more stringent construct incorporating objective physiological and inflammatory parameters. However, this definition is not universally accepted. In analogy with other chronic inflammatory diseases, it may be more appropriate to consider clinical remission and biological remission as complementary but distinct dimensions. In asthma, the degree to which clinical stability reflects true biological quiescence remains to be fully elucidated.

A critical issue emerging from the available literature is the marked heterogeneity in the elements used to define remission, particularly regarding symptom thresholds and lung function. Several studies adopt an ACQ cut‐off of < 1.5, which reflects well‐controlled asthma rather than true absence of symptoms. In contrast, a more stringent threshold (e.g., ACQ < 0.75) may better represent minimal or absent symptom burden. The use of higher cut‐offs raises the concern that some current definitions of remission may in fact capture sustained good control rather than a truly robust remission state.

Lung function represents one of the most controversial components. Some definitions require normalization of lung function (e.g., post‐bronchodilator FEV1 ≥ 80% predicted), whereas others accept stability or minimal decline over time, acknowledging the presence of fixed airflow limitation in patients with long‐standing or severe disease. While stabilization may represent a meaningful therapeutic objective in such contexts, it raises the conceptual question of whether absence of progression alone is sufficient to define remission. On the other hand, improvement in pulmonary function should be interpreted primarily as a marker of treatment response or remission induction rather than a mandatory criterion of remission itself.

An additional conceptual challenge is that some remission definitions, especially in the context of biologic therapies, may in practice correspond to a state of excellent disease control rather than a stringent remission state. In certain definitions, remission is determined without fully considering the persistence of background maintenance therapy, reliance on reliever medication, or broader patient‐reported outcomes. This suggests that, in some instances, proposed remission criteria may approximate optimized asthma management rather than a distinct disease state, underscoring the importance of clearly distinguishing remission from high‐level control.

## 3 Asthma Remission Predictors

Several baseline characteristics have been identified as positive predictors of spontaneous asthma remission [6]. These include a younger age of asthma onset, milder symptoms at the time of diagnosis, male sex, and higher baseline lung function [10, 20, 21, 22, 23]. Additionally, lower levels of bronchial hyperresponsiveness, reduced blood eosinophils, and allergen sensitisations are also associated with a greater likelihood of remission [23, 24, 25]. Patients without comorbidities such as nasal polyps, eczema, atopy, or rhinitis, as well as those with no history of pneumonia, a negative family history of asthma and atopy, and who have quit smoking, are more likely to experience asthma remission [20, 23, 25, 26, 27, 28]. Predictors of asthma remission under biologics will be discussed in Section 8.

## 4 Airway Inflammation and Epithelial Damage in Asthma Remission

The airway epithelium is a key coordinator of immune responses, since it is the interface with the external environment and it is the first line of defense against allergens, pathogens, and environmental toxins. In asthmatic patients, after epithelial barrier disruption “alarmins” (TSLP, IL‐25, and IL‐33) are released and promote both innate and adaptive T2 immune responses [29]. IL‐25 and IL‐33 activate T2 innate lymphoid cells to produce IL‐5 and IL‐13, promoting T2 inflammation [30]. TSLP is a potent activator of mast cells, triggering the release of T2 cytokines including IL‐5 and IL‐13 [31]. Epithelial damage also triggers the release of fibrogenic cytokines such as IL‐1β, IFN‐γ, TNF‐α, IL‐4, and IL‐13. IFN‐γ, TNF‐α, and T‐helper 17 cytokines drive non‐T2 asthma endotype [32, 33, 34]. This complex immunological misregulation leads to barrier disruption, immune hyperreactivity, mucus hypersecretion, mucus plugging, airway hyperresponsiveness (AHR), and finally to tissue remodeling. All these mechanisms might be changed in patients who achieve remission, and one of the aims of modern research is to validate predictive biomarkers of disease course.

At present, some biomarkers are related to airway inflammation and epithelial damage and can help the clinicians to define disease control and “biologic” or “inflammatory” remission. Fractional exhaled nitric oxide (FeNO) levels, blood and sputum eosinophils count reflect T2 driven airway inflammation [35, 36] and biologics targeting downstream effectors of inflammation such as IL‐4, IL‐5, IL‐13, and IgE are effective for T2 patients. In eosinophilic asthma, mepolizumab has shown a potential disease‐modifying effect: in the REMOMEPO study [37], reduction in airway inflammation was associated with decreased bronchial remodeling, while in the MESILICO study [38], 12 months of treatment led to clinical and functional improvement, reduced exacerbations and a significant regression of multiple remodeling indices (including epithelial damage), correlated with the reduction in tissue eosinophils.

In contrast, non‐T2 or T2‐low patients—who typically have low baseline blood eosinophils (BEC < 150/mmc) and low FeNO levels (FeNO < 25 ppb) —are less likely to benefit, as the mechanisms underlying their asthma are poorly understood and not currently targetable by monoclonal antibodies; available evidence suggests that alarmin‐targeting monoclonal antibodies including Tezepelumab act early in the inflammatory cascade and reduce exacerbations in T2‐low asthma (PATHWAY and NAVIGATOR pooled analysis); however, data on biomarkers and clinical remission remain lacking [39]. Currently, the absence of T2 inflammation hinders the achievement of asthma remission. However, targeting non–T2‐driven traits as risk factors and comorbidities (e.g., obesity treatment, including GLP‐1 and gastric inhibitory polypeptide receptor agonists [40]) may be beneficial; moreover, add‐on therapies as Azithromycin potentially targets microbial dysbiosis in T2‐low asthma [40]. Emerging therapies are listed in section dedicated to Future perspectives.

According to the Delphi consensus conducted by the Severe Asthma Network in Italy (SANI), inflammatory remission is characterized by a low concentration or absence of inflammatory markers (such as eosinophils, allergen‐specific IgE, periostin, FeNO, eventually airway obstruction); the cutoffs of eosinophil value < 300 cells/L and of FeNO < 25 ppb have been defined as good markers for reduction in inflammation, but a shared criterion for inflammatory remission is still lacking [18, 29].

The use of biomarkers across the studies included in the review is heterogeneous and only rarely incorporated into definitions of remission. In particular, Pavord et al. [41] included biomarkers within the definition of deep remission, defined as clinical remission combined with the assessment of FeNO, blood eosinophil count (BEC), and total IgE levels. Conversely, Oishi et al. [42] introduced immunological remission (BEC < 300 cells/mm^3^ and FeNO < 35 ppb after 12 months of biologic therapy), an integral part of deep remission along with clinical and functional remission; clinical, immunological, and deep remission were observed in 68.5%, 50%, and 31.5% of patients, respectively. Type 2 inflammation may be fully suppressed by biologics, but has been quantified only in a few studies defining inflammatory remission.

Similarly, some studies conducted with tezepelumab defined biological remission, characterized by BEC < 150 cells/mm^3^ [43] or < 300 cells/mm^3^ [44] and FeNO < 25 ppb. According to Wechsler et al. [43], the biologic remission rate was 33% after 24 months of biologic therapy, and complete remission was defined by the concomitant achievement of clinical and biological remission; however, these domains remain distinct, as 19% of patients in clinical remission still exhibited BEC ≥ 150 cells/mm^3^ and FeNO ≥ 25 ppb. In a separate analysis by Gates et al. [43], only 40% of patients in biological remission achieved clinical remission, while 42.9% of those in clinical remission also reached biological remission; in this study, the biologic remission rate at 12 months was 38%.

Although many authors agree on the utility of biomarkers in defining asthma remission and in predicting accelerated lung function decline, it should be borne in mind that asthma control is complex and also depends on infectious exacerbations and exposure to environmental factors (i.e., tobacco smoking) [45, 46, 47].

However, Rogers et al. [48] evaluated a significant reduction in biomarkers as FeNO, BEC and total IgE level also in children and adolescents treated with biologics. The highest decrease of FeNO was associated to Dupilumab and Benralizumab therapy, BEC decrease was more significant with Mepolizumab and Benralizumab and a decrease in total IgE level was observed for Dupilumab and Tezepelumab.

Currently, biomarkers are mainly used as predictive values for choosing the right biologic therapy; data coming from Liberty Venture [49] and Liberty Quest [50] for Dupilumab, MENSA [51] and SIRIUS [52] studies for Mepolizumab, SIROCCO [53], CALIMA [54] and ZONDA [55] studies for Benralizumab, NAVIGATOR [56] and SOURCE [57] for Tezepelumab, show us that patients with higher FeNO levels can benefit from Dupilumab, Mepolizumab and Tezepelumab, but those with high blood eosinophils count (BEC) can improve with Dupilumab, Mepolizumab, Reslizumab, Benralizumab and Tezepelumab.

Studies reporting significant biomarker findings in asthma remission are summarized in Table 1 and show substantial heterogeneity; consequently, the role of individual biomarkers in remission remains unclear.

**TABLE 1:** Overview of biomarkers with statistically significant results measured in clinical asthma remission in the studies included in this review.

Biologic agent	First author, year	BEC (cell/mmc) trend in CR	FeNO (ppb) trend in CR	Total IgE (UI/mL) trend in CR	Other biomarkers in CR	Evaluation period (months)
Anti IL‐4Ra monoclonal antibody (Dupilumab)	Portacci et al. (2023) [58]	Mean BEC ↑ (570) in CR	N.S.	—	—	12
	Gates et al. (2024) [59]	—	Higher bFeNO*** is associated to CR	N.S.	—	24
	Bagnasco et al. (2025) [60]	N.S.	bFeNO ↓ in CR (mean FeNO: 44 → 24)	bIgE**** ↓ in CR (mean value 508 → 133)	—	12
	Pavord et al. (2025) [41]	*Traverse*: uniform median BEC from baseline (330 → 280) in CR	*Traverse*: Median bFeNO ↓ in CR: 30 → 16	—	—	12
Anti‐IL‐5 monoclonal antibody (Mepolizumab)	Pavord et al. (2023) [61]	Higher mean bBEC* (> 700) is more common in CR	—	—	—	12
	Bagnasco et al. (2024) [62]	Higher bBEC is favorable predictor of 36 months CR	Mean bFeNO < 46 associated to 12 months CR	—	—	12–36
Anti‐IL‐5 monoclonal antibody receptor (Benralizumab)	Menzies‐Gow et al. (2022) [63]	*SIROCCO*: – bBEC ≥ 150: 15.9%** – bBEC ≥ 300: 16.2%** *CALIMA*: – bBEC ≥ 150: 15.7%** – bBEC ≥ 300: 17.5%** *ZONDA*: – bBEC ≥ 150: 22.5%** – bBEC ≥ 300: 22.2%**	—	—	—	6
		*SIROCCO*: – bBEC ≥ 150: 13.7%** – bBEC ≥ 300: 15.6%** *CALIMA*: – bBEC ≥ 150: 16.9%** – bBEC ≥ 300: 18%**	—	—	—	12
	Numata et al. (2022) [64]	Mean bBEC ↓ in ↑ pulmonary function (605 → 43); no data in CR	bFeNO ≥ 50 in patients with ↓ exacerbations (no statistical significance in CR)	—	—	24
	Martinez‐Moragon et al. (2024) [65]	bBEC ≥ 300 have lower FeNO at 12 months (data on CR not significant)	FeNO ↓ is associated to improved Quality of Life (no data on CR)	—		12
Anti‐TSLP monoclonal antibody (Tezepelumab)	Wechsler et al. (2024) [43]	Mean bBEC is 366 and 406 in CR and complete remission, respectively → higher bBEC is associated to complete remission	bFeNO higher in CR (mean value 43.2); mean FeNO: 47.7 (over 0–52 weeks) is associated to complete remission	Mean bIgE is 557.7 in CR and mean IgE is 514.4 kU/L over 0–52 weeks in complete remission	—	12
	Gates et al. (2025) [44]	Median bBEC is 460 and ↓ < 300 in CR	Median bFeNO is 70 and ↓ < 25 in CR	Median bIgE is 189 in CR	—	12
	Sumi et al. (2025) [66]	BEC uniform despite clinical improvement	FeNO unchanged despite clinical improvement	Mean bIgE ↓ in patients treated; responders' bIgE is 74	—	6
Multiple biologics						
Mepolizumab, Reslizumab	Moermans et al. (2023) [67]	– BEC < 300 is required for remission – Mean bBEC is not statistically significant	FeNO is similar in CR and non remission	bIgE higher in CR	– Baseline sputum eosinophil, macrophage and lymphocyte counts markedly elevated – Baseline sputum eotaxin‐1, TSLP, IL‐5, EPX and IgE protein levels are higher	12
Omalizumab, Mepolizumab, Benralizumab and Dupilumab	Oishi et al. (2023) [42]	N.S.	N.S.	N.S.	—	12
Omalizumab, Mepolizumab, Benralizumab and Dupilumab	Sposato et al. (2023) [68]	N.S.	Mepolizumab: higher bFeNO is favorable predictor for CR (mean bFeNO is 76.2 in CR)	N.S.	—	12
Omalizumab, Mepolizumab, Reslizumab, Benralizumab and Dupilumab	Milger et al. (2023) [14]	—	—	N.S.	—	12
Omalizumab, Mepolizumab, Reslizumab, Benralizumab and Dupilumab	McDowell et al. (2023) [69]	Mean bBEC 400 in CR	Mean bFeNO is 51 in CR	—	*Composite T2 status*: High BEC (≥ 150/mm^3^)/High FeNO (≥ 20 ppb); possible predictor of remission (74.8% of remitters)	12
Mepolizumab, Benralizumab	Carpagnano et al. (2024) [70]	Higher bBEC is predictor for CR	—	—	—	12
Omalizumab, Mepolizumab, Reslizumab, Benralizumab and Dupilumab	Hansen et al. (2024) [71]	Mean bBEC is 500 in CR for anti‐IL5/IL‐5R therapy → predictor of CR	– Omalizumab: mean bFeNO is 7 in CR – Dubilumab: mean bFeNO is 52 in CR; bFeNO predictor of CR (for Dupilumab and Omalizumab)	Anti‐IL5/IL5R therapy: mean bIgE is 205 in CR; bIgE predictor of CR	—	12
Benralizumab, Mepolizumab, Dupilumab, Omalizumab, Reslizumab	Chipps et al. (2024) [72]	Mean bBEC is 479.2 in CR	Mean bFeNO is 45.2 in CR	Mean bIgE is 421.3 in CR	—	12–48
Mepolizumab, Benralizumab, Reslizumab	Bal et al. (2025) [73]	Mean bBEC is 500 in CR	bFeNO significantly ↓ in CR	N.S.	—	12–72
Mepolizumab, Benralizumab, Reslizumab	Blouin et al. (2025) [74]	bBEC ≥ 300 is predictor of CR (median bBEC in CR is 775)	—	—	*Median baseline sputum eosinophils*: 25.9% (higher vs. non‐remitters)	12
Omalizumab, Mepolizumab, Benralizumab, Dupilumab	Canonica et al. (2024) [75]	Median bBEC is 500 in CR	Median bFeNO is 55.6 in CR	Median bIgE is 177.5 in CR	—	12
Omalizumab, Mepolizumab, Benralizumab, Dupilumab	Cosini et al. (2025) [76]	Higher bBEC is associated to complete asthma remission (mean bBEC is 953 in remission)	—	—		> 24

*Note:* All remission outcomes were compared with patients who did not achieve remission or with the overall study population.

Abbreviations: —, not evaluated; %**, the value is referred to the percentage of patients who achieved remission; *bBEC, baseline eosinophils count; BEC, blood eosinophils count; bFeNO***, baseline fraction exhaled nitric oxide; bIgE****, baseline total IgE; CR, clinical remission; FeNO, fraction exhaled nitric oxide; N.S., not statistically significant.

## 5 Bronchial Obstruction in Asthma Remission

Bronchial obstruction in asthma appears to be the result of multiple interacting mechanisms, including bronchial constriction, airway inflammation, mucus impaction and remodeling. Epithelial cytokines in asthma act on myofibroblasts and airway smooth muscle (ASM) cells, leading to airway hyperreactivity. Structural changes observed in asthmatic airways include ASM cell hypertrophy, hyperplasia and hypercontractility [29]. Increased expression of ECM components within the ASM layer has been shown to correlate with airway change of function (FEV1 reversibility) in patients with asthma, especially if severe [77]. Imbalances in the disposition and degradation of subepithelial ECM can lead to subepithelial fibrosis, airway wall thickening and potentially irreversible airflow obstruction, with consequent lack of response to treatment. Moreover, in chronic bronchoconstriction, increased mucus secretion can lead to mucus plugging, a phenomenon observed in patients with fatal asthma [78, 79, 80]. Therefore, lung function should be evaluated individually, considering also imaging findings and relevant anatomical factors (e.g., airway remodeling, mucus plugs), with the aim of stabilization and preventing further decline. Until recently, biologic therapies were thought to improve lung function primarily by targeting T2‐driven inflammation, with limited impact on airflow limitation related to structural airway damage. However, emerging evidence suggests that some agents as mepolizumab may exert disease‐modifying effects in eosinophilic asthma: reduced airway inflammation was associated with decreased bronchial remodeling in the REMOMEPO study [37], while 12 months of treatment led to clinical and functional improvement, and regression of remodeling indices ‐as epithelial damage bioptically documented‐ in the MESILICO study [38].

Bronchoconstriction can be quantified measuring FEV1 at different stages of asthmatic disease and during specific treatments. Most authors approve to measure FEV1 before and after at least 6–12 months from the beginning of biologic treatment to indirectly monitor airway remodeling, but there is no consensus about the entity of improvement in lung function neither in Delphi consensus conducted by the Severe Asthma Network Italy (SANI) [18], neither in the German Asthma Net severe asthma registry cohort study [14] Afterall, the well‐known intraindividual variability of spirometry values might itself hamper the accuracy of lung function assessment, whose relevance should be contextualized in the frame of other variables. Some studies evaluated the improvement in pre‐bronchodilator FEV1 (mL), globally varying from 90 to 159 mL: LIBERTY QUEST and LIBERTY VENTURE for Dupilumab [49, 50], MENSA [51] and SIRIUS [52] studies for Mepolizumab, SIROCCO [53], CALIMA [54] and ZONDA [55] studies for Benralizumab, NAVIGATOR [56] and SOURCE [57] for Tezepelumab. However, there is no universal consensus on the “value to reach” in induction of remission, and recently the use of the FEV1 improvement of more than or equal to 100 mL as induction of remission criterion was criticized as too low [9, 63, 81].

The damage to the airway related to chronic inflammation can be irreversible and some authors consider sufficient for remission a FEV1 ≥ 80% after 1 year of biologic treatment, in union to the other parameters used to define remission [70]. A post‐bronchodilator FEV1 ≥ 80% of predicted is considered sufficient for clinical remission also in the REDES study [61], in an observational multicenter retrospective study published by Maglio et al. [82] and according to Menzies‐Gow et al. [9] complete remission would include also negative bronchial hyperresponsiveness tests or degree of epithelial fibrosis (epithelial thickness).

Airway inflammation in remission has already been discussed in the previous section.

As far as concerns mucus plugging, Tezepelumab is the first biologic that has demonstrated the ability to reduce it [83] but recently also the other biologics have shown promising results among severe asthmatics patients: Dupilumab (in the VESTIGE study [84]), Benralizumab [85, 86] and Mepolizumab [87] reduce the mucus plug score from baseline in HRCT; moreover, Mepolizumab and Benralizumab therapy are associated with improvement in inflammatory biomarkers and clinical outcomes, even if their influence on remission is not statistically significant [87] or has not been studied [86].

All biologicals now available have demonstrated to improve airway remodeling through different mechanisms: while Omalizumab and Mepolizumab reduce basal membrane thickness (and respectively also ECM deposition and density) [88, 89, 90, 91], Benralizumab and Dupilumab improve airway hyperreactivity to mannitol over 12 weeks in patients with T2‐asthma [92, 93] and Dupilumab reduces goblet cell metaplasia (in murine model) [94]. Some clinical trials are now ongoing in order to analyze the relationship between patients treated with Benralizumab and airway remodeling; they include BENRAMOD (NCT04365205) [95] and CHINOOK (NCT03953300) [96].

Airway remodeling was also evaluated by performing bronchoscopy biopsies in moderate‐to‐severe asthma patients during Tezepelumab therapy, in the phase II CASCADE study [97]. At present, however, it is not possible to establish a correlation between airway remodeling and asthma remission, as remodeling represents only one of the complex mechanisms involved in asthma.

In conclusion, all components of bronchial obstruction are potentially reversible with biologic therapies, albeit through different mechanisms. However, given the multifactorial nature of bronchial obstruction, comprehensive and differential assessment of all contributing factors remains challenging. Accordingly, reversibility is likely more difficult—or even unattainable—in long‐standing and poorly controlled disease.

## 6 Unmet Needs in Definition of Asthma Remission

There are many unmet needs in the definition of asthma remission. First, a universally shared definition distinguishing clinical, inflammatory, and complete remission is still lacking. Secondly, if we consider complete remission as the complete absence of symptoms and airway function restored [17], the distinction between remission and true recovery from asthma remains unclear. These open questions are challenging and still under investigation; moreover, prospective evidence is currently absent and generally limited regarding how many patients and under which conditions (disease severity, treatment, and duration) may achieve remission.

A critical appraisal of remission definitions is therefore required, given the substantial heterogeneity of parameters adopted across studies. In some reports, remission is defined solely by the absence of exacerbations and by symptom control assessed using ACT or ACQ scores, with no agreement on the minimum score required. Other authors consider a partial improvement in airway remodeling sufficient, even when pre‐bronchodilator FEV1 improvement is < 100 mL, regardless of the percentage of predicted FEV1. Anyway, the majority of studies including functional outcomes converge on a post‐bronchodilator FEV1 ≥ 80% of predicted as a key criterion for remission. Moreover, important data as biomarkers (i.e., FEV1 reversibility, blood sputum eosinophils count and FeNO in T2‐patients), quality of patients' lives and maintenance of remission without OCS and/or reduced dose of ICS, are frequently not assessed.

Since eosinophilic asthma is the most common asthma phenotype, complete remission should encompass clinical remission criteria together with biomarker normalization and resolution of bronchial hyperresponsiveness [9]. In the coming years, tapering of ICS dosage may reasonably be incorporated into remission criteria, supported by encouraging data from studies with Benralizumab showing a sustained probability of ICS dose reduction [98, 99].

In most studies, the duration required to determine remission is at least 12 months, but persistent remission can be obtained only after 3 years, according to the majority of the Delphi consensus conducted by the Severe Asthma Network Italy [18]. It is also important to note that none of the studies assessed treatment‐induced remission in mild and moderate asthma, which identifies a significant knowledge gap that needs to be addressed. A study from a multicenter survey in Japan evaluated clinical remission in asthma patients treated with ICS/LABA without biologics, defining remission at increasing levels of stringency. It found that 56.9% met criteria for 3‐way clinical remission (no exacerbations, no OCS, ACQ‐5 < 1.5), 35.0% met 4‐way remission (adding normal lung function), and 24.7% achieved deep remission (including low FeNO). Physician–patient discordance in asthma control perception was a significant barrier to achieving remission [100]. Data now available examine mainly patients with severe asthma who are receiving biologics, but there's no consensus about either discontinuation of biologic treatment or its maintenance and whether asthma could be completely controlled with biologic treatments. On this matter, criteria suggested by Delphi Italian consensus should be applied to patients with severe asthma on treatment with biologic drugs, and there are no data of remission in patients off treatment [18].

## 7 The Super Responder Model

Upham et al. [101] developed an international consensus‐based definition for severe asthma super‐responders, using a modified Delphi process. The definition comprised improvements in three domains assessed over 12 months, including improvement in at least two of the following major criteria: exacerbation elimination, a large improvement in asthma control and cessation of long‐term OCS; it also included improvement in one of the following minor criteria: 75% exacerbation reduction, 500 mL or greater improvement in FEV1, or achievement of well‐controlled asthma. Criteria for clinical remission are broadly consistent with those for super‐response, but remission represents an important step beyond super‐response, eliminating exacerbations and asthma symptoms for a sustained period. However, patients with less active disease could be considered super‐responders, while not meeting the stricter definition of clinical remission [9, 101].

Some recent studies have identified severe asthma super‐responders after at least 2 years of treatment with all anti‐IL‐5 drugs (Mepolizumab, Benralizumab, Reslizumab) [102, 103, 104], but they did not use the composite outcome remission: the RELIght study, a real‐world study in which patients with severe eosinophilic asthma were treated with Mepolizumab, has focused on assessing a patient's level of treatment response to identify responders and super‐responders [105].

The outcome of “super responders” is not assessed in many of the currently available studies on remission; therefore, at present, it is advisable to refer solely to the term remission in order to avoid conceptual ambiguity. Although positive data on Omalizumab discontinuation in super‐responder patients in clinical remission are available [106], this approach is currently limited to small populations and is not generalizable.

## 8 Remission of Severe Asthma With Biologics

Over the past decade, an increasing understanding of the underlying biology of asthma has led to the development of new treatment targets. The management of severe asthma has been revolutionized by emerging biologic treatments, with more optimistic therapeutic goals. It is crucial to note that the therapeutic effects of biologics are significantly influenced by the presence of active type 2 inflammation. In patients with severe airways disease and elevated biomarkers, the treatment benefits of biologics are three‐ to five‐fold greater than in patients without type 2 inflammation [107, 108, 109]. Biologics that target TH2 cytokines include anti‐IL‐4R (Dupilumab), anti‐IL‐5 (Mepolizumab, Reslizumab) and anti‐IL‐5R (Benralizumab). Besides Tezepelumab is a human mAb that blocks thymic stromal lymphopoietin, an epithelial‐cell derived cytokine implicated in the pathogenesis of asthma [1].

For clinicians, one of the significant goals of the severe asthma treatment is achieving a clinical remission of the disease. Many clinical trials (RCTs) and real‐life studies of biologics have addressed the concept of clinical remission and reported encouraging results [14, 41, 42, 43, 44, 58, 59, 60, 61, 62, 63, 64, 65, 66, 67, 68, 69, 70, 71, 72, 73, 74, 75, 76, 82, 109, 110, 111, 112, 113, 114, 115, 116, 117, 118, 119, 120, 121, 122, 123, 124, 125, 126, 127, 128, 129, 130, 131]. However, a recent systematic review and meta‐analysis by Shackleford et al. showed that clinical remission in severe asthma patients treated with biologics was achieved in 30%–38% of cases, depending on the definition used, highlighting significant variability across studies. Factors such as longer disease duration, poorer lung function, higher symptom burden, and comorbidities like obesity and depression were consistently associated with lower remission rates [132]. Studies evaluating clinical remission with biologicals were summarized in detail in Table 2. Studies evaluating predictors of clinical remission with biologics are summarized in detail in Table 3. This section summarizes the criteria used in the studies conducted for each biologic.

**TABLE 2:** Overview of biologic therapies and key parameters used to define asthma remission in the studies included in this review.

Biologic agent	First author, year	Study design	Symptoms	Lung function	No exacerbations	No OCS	Clinical remission rate (%)	Evaluation period (months)
Anti IgE monoclonal antibody (Omalizumab)	Cilli et al. (2024) [110]	Retrospective	ACQ‐6 ≤ 1 and/or ACT ≥ 20	Pre‐BD FEV1 of ≥ 80% predicted	✓	✓	25.9%	12
Anti IL‐4Ra monoclonal antibody (Dupilumab)	Douglass et al. (2022) [112]	Post‐hoc analysis	ACQ‐5 < 1.5	Post‐BD FEV1 ≥ 80% OR improvement in pre‐BD FEV1 ≥ 100 mL	✓	✓	31.7%–36.4%	11
	Pelaia et al. (2023) [113]	Retrospective	ACT ≥ 20	FEV1 ≥ 80% of predicted value	✓	✓	47.2%	6
	Portacci et al. (2023) [58]	Prospective	ACT ≥ 20	FEV1 improvement ≥ 100 mL from baseline	✓	✓	38.9%	12
	Bult et al. (2024) [114]	Retrospective	ACQ‐5 < 1.5	FEV1 ≥ 80% of predicted value and/or a change in FEV1 ≥ 100 mL	✓	✓	31.6%	12
	Quarato et al. (2024) [115]	Retrospective	ACT ≥ 20	Pre‐BD FEV1 ≥ 80% of the predicted value	✓	✓	30%	12
							45%	24
	Gates et al. (2024) [59]	Retrospective	ACQ‐6 < 1.5	Stable lung function	✓	✓	43%	12
	Bagnasco et al. (2025) [60]	Retrospective	ACT ≥ 20	FEV1 stabilization	✓	✓	45%	12
	Pavord et al. (2025) [41]	Post‐hoc analysis	ACQ‐5 < 1.5	A decline from parent study baseline in pre‐ or post bronchodilator FEV1 by no more than 5%	✓	✓	49.4%	3
							48%	6
							37.2%	12
							46.2%	18
							42.8%	24
	Al‐Ahmad et al. (2025) [116]	Retrospective	ACT ≥ 20 and ACQ‐6 ≤ 1.5	Predicted forced expiratory volume in 1 s (FEV1%) ≥ 80%	✓	✓	18.1%	12
Anti‐IL‐5 monoclonal antibody (Mepolizumab)	Pavord et al. (2023) [61]	Post‐hoc analysis	ACT ≥ 20	Postbronchodilator FEV1 ≥ 80%	✓	✓	30%	12
	Bagnasco et al. (2024) [62]	Retrospective	ACT ≥ 20	Improved value or a maximal decline from a previous test of 150 mL	✓	✓	39%–52%	12
	Cheng et al. (2025) [119]	Retrospective	ACT ≥ 20	FEV1 change from baseline ≥ 0 mL or FEV1 improvement from baseline ≥ 100 mL or ≤ 100 mL decrease from baseline in FEV1	✓	✓	23%	12
	Hamada et al. (2025) [120]	Retrospective	ACQ‐5 ≤ 1	FEV1% predicted greater than or equal to 80% and/or not greater than a 5% decline from baseline	✓	✓	30.6%	12
	Di Bona et al. (2025) [121]	Retrospective	ACT ≥ 20	Postbronchodilator FEV1 ≥ 80%	✓	✓	9.9%–68.3%	12
	Papaioannou et al. (2025) [122]	Post‐hoc analysis	ACT ≥ 20	Stable or improved lung function	✓	✓	27.4%	12
							22%	24
	Hamada et al. (2025) [123]	Retrospective	ACQ‐5 ≤ 1.0	Post‐BD FEV1 ≥ 80% or Post‐BD FEV1 < 5% decline from baseline	✓	✓	5.7%–36.5%	12
	Correa‐Borit et al. (2025) [124]	Retrospective	ACT ≥ 20	Not included	✓	✓	58%	36
							63%	48
Anti‐IL‐5 monoclonal antibody receptor (Benralizumab)	Menzies‐ Gow et al. (2022) [63]	Post‐hoc analysis	ACQ‐6 < 1.5 or ≤ 0.75	Pre‐BD FEV1 increase ≥ 100 mL	✓	✓	14.5%	12
	Harrison et al. (2022) [117]	Post‐hoc analysis	ACQ‐6 < 1.5″ or ≤ 0.75	Pre‐BD FEV1 increase ≥ 100 mL	✓	✓	16.6%–28.7%	6
	Numata et al. (2022) [64]	Retrospective	ACT ≥ 20	FEV1% ≥ 80%	✓	✓	21.7%	24–48
	Jackson et al. (2023) [118]	Post‐hoc analysis	ACQ‐5 < 1.5 or ACT ≥ 16	Not included	✓	✓	43%	≥ 12
	Padilla‐Galo et al. (2023) (ORBE II) [125]	Post‐hoc analysis	ACT ≥ 20	Pre‐BD FEV1 increase ≥ 100 mL	✓	✓	43.7%	12
	Martinez‐Moragon et al. (2024) [65]	Retrospective	ACT ≥ 20	Pre‐ BD FEV1 of ≥ 80%	✓	✓	44.1%	12
	Pini et al. (2024) [126]	Retrospective	ACT ≥ 20	Pulmonary function stability	✓	✓	69.5%	12
	Quarato et al. (2025) [127]	Retrospective	ACT score ≥ 20	Pre‐bronchodilation FEV1 ≥ 80% predicted	✓	✓	30.4%	12
							52.1%	24
							65.2%	48
Anti‐TSLP monoclonal antibody (Tezepelumab)	Wechsler et al. (2024) [43]	Post‐hoc analysis	ACQ‐6 ≤ 1.5	FEV1 > 95% of baseline at the end of each year	✓	✓	28.5%	12
	Gates et al. (2025) [44]	Retrospective	ACQ‐6 ≤ 1.5	Stable lung function	✓	✓	36%	12
	Sumi et al. (2025) [66]	Retrospective	ACT ≥ 23	Not included	✓	✓	18%	6
Multiple biologics								
Omalizumab, Mepolizumab	Yesilkaya et al. (2024) [128]	Retrospective	ACT ≥ 20	Improvement of > 10% from baseline FEV1%	✓	✓	72.9%	12
Mepolizumab, Benralizumab	Maglio et al. (2023) [82]	Retrospective	ACT ≥ 20	Post‐BD FEV1 ≥ 80%	✓	✓	30.12%–40%	12
Mepolizumab, Reslizumab	Moermans et al. (2023) [67]	Retrospective	ACQ < 1.5 and or ACT > 19	FEV1 ≥ 80% and/or improvement of FEV1 ≥ 10% and blood eosinophil count < 300 cells/mL	✓	✓	21.1%	12
Omalizumab, Mepolizumab, Benralizumab and Dupilumab	Oishi et al. (2023) [42]	Retrospective	ACQ < 1.5	FEV1 ≥ 80% of predicted	✓	✓	68.5%	12
Omalizumab, Mepolizumab, Benralizumab and Dupilumab	Sposato et al. (2023) [68]	Retrospective	ACT ≥ 20	FEV1 ≥ 80% of predicted	✓	✓	21.8%–35.8%	12
Omalizumab, Mepolizumab, Reslizumab, Benralizumab and Dupilumab	Milger et al. (2023) [14]	Retrospective	ACT ≥ 20	Pre‐BD FEV1 increase ≥ 100 mL	✓	✓	32.1%	12
Omalizumab, Mepolizumab, Reslizumab, Benralizumab and Dupilumab	McDowell et al. (2023) [69]	Retrospective	ACQ‐5 < 1.5	FEV1 > lower limit of normal or < 100 mL less than baseline	✓	✓	18.3%	12
Omalizumab, Mepolizumab	Thomas et al. (2024) [129]	Retrospective	ACQ‐5 ≤ 1	Post‐BD FEV1 ≥ 80% or Post‐BD FEV1 < 5% decline from baseline	✓	✓	19.1%–25.2%	12
Mepolizumab, Benralizumab	Carpagnano et al. (2024) [70]	Retrospective	ACT ≥ 20	FEV1 ≥ 80%	✓	✓	30.5%	12
Omalizumab, Mepolizumab, Reslizumab and Benralizumab	Valverde‐Monge et al. (2024) [130]	Retrospective	ACT > 20	Pre‐BD FEV1 > 80%	✓	✓	27%	12
Omalizumab, Mepolizumab, Reslizumab, Benralizumab and Dupilumab	Hansen et al. (2024) [71]	Prospective	ACQ‐6 ≤ 1.5FEV1 > 80% of predicted value	FEV1 > 80% of predicted value	✓	✓	19%	12
Omalizumab, Mepolizumab, Reslizumab, Benralizumab and Dupilumab	Breslavsky et al. (2024) [131]	Retrospective	ACT ≥ 20, ACQ6 < 1.5, ACQ6 ≤ 0.75	FEV1% ≥ 80%, FEV1/FVC ≥ 0.75, improvement of FEV1 ≥ 10% from baseline, improvement of FEV1 ≥ 100 mL from baseline	✓	✓	20%–41%	12
Benralizumab, Mepolizumab, Dupilumab, Omalizumab, Reslizumab	Chipps et al. (2024) [72]	Retrospective	ACT score ≥ 20	Not included	✓	✓	22.3%	12–13
							34.3%	47–48
Mepolizumab, Benralizumab, Reslizumab	Bal et al. (2025) [73]	Retrospective	ACT score ≥ 20	Not included	✓	✓	35.7%	12–72
Mepolizumab, Benralizumab, Reslizumab	Blouin et al. (2025) [74]	Retrospective	Good asthma control	≤ 10% decrease in pre‐bronchodilator FEV1 compared with baseline	✓	✓	28.6%	12
Omalizumab, Mepolizumab, Benralizumab, Dupilumab	Canonica et al. (2024) [75]	Retrospective	ACT ≥ 20 or ACQ < 1.5	FEV1 decline < 100 mL over 12 months	✓	✓	45.8%	12
Omalizumab, Mepolizumab, Benralizumab, Dupilumab	Cosini et al. (2025) [76]	Retrospective	ACT score ≥ 20	FEV1 ≥ 80%	✓	✓	41%	> 24

Abbreviations: ACQ, Asthma Control Questionnaire; ACT, Asthma Control Test; BD, Bronchodilator; FEV1, Forced Expiratory Volume in 1 s; FEV1/FVC, Ratio of Forced Expiratory Volume in 1 s to Forced Vital Capacity; OCS, Oral Corticosteroids; Post‐BD FEV1, Forced Expiratory Volume in 1 s measured after bronchodilator administration; Pre‐BD FEV1, Forced Expiratory Volume in 1 s measured before bronchodilator administration.

**TABLE 3:** Overview of predictors of in clinical asthma remission in the studies included in this review.

Biologic agent	First author, year	Predictors of clinical remission	Evaluation period (months)
Anti‐IL‐4Ra monoclonal antibody (Dupilumab)	Bult et al. (2023) [114]	Baseline blood eosinophil counts (BEC) > 300/μL	12 months
		Male sex	
	Bagnasco et al. (2025) [60]	Aspirin‐exacerbated respiratory disease	12 months
		Concomitant CRSwNP	
Anti‐IL‐5 monoclonal antibody (Mepolizumab)	Bagnasco et al. (2024) [62]	FEV1	12 months
		FEV1 and higher number of eosinophils	36 months
	Hamada et al. (2024) [120]	ACQ‐5 score at both 3 and 6 months	12 months
		OCS dose at both 3 and 6 months	
		Exacerbation frequency	
		Lower OCS dose	
		Greater forced expiratory volume in 1 s percentage predicted	
		Higher Asthma Quality of Life Questionnaire score	
		Higher OCS dose	
		Nasal polyps	
Anti‐IL‐5 monoclonal antibody receptor (Benralizumab)	Quarato et al. (2025) [127]	Nasal polyposis	48 months
		Osteoporosis	
		OCS dependency	
		Predicted pre‐bronchodilation FEV1% ≥ 80% at baseline	
		Predicted FEF25%–75% < 65% at baseline	
Anti‐TSLP monoclonal antibody (Tezepelumab)	Gates et al. (2025) [44]	Female gender	12 months
		Lower BMI	
		Adult‐onset asthma	
		Higher FeNO	
		Lower ACQ6	
Multiple biologics			
Mepolizumab, Reslizumab	Moermans et al. (2023) [67]	Male sex	12 months
		Sputum neutrophil percentage, eotaxin‐1, IL‐5, and eosinophil peroxidase	
Omalizumab, Mepolizumab, Benralizumab, and Dupilumab	Oishi et al. (2023) [42]	Asthma duration ≤ 5 years	12 months
		FEV1 ≥ 75% predicted	
Omalizumab, Mepolizumab, Benralizumab, and Dupilumab	Sposato et al. (2023) [68]	For omalizumab, younger age, lower BMI, an earlier age of asthma onset, absence of sinusitis/nasal polyposis absence of hypertension and chronic heart disease and lower exacerbation numbers	12 months
		For mepolizumab, higher FeNO	
		For benralizumab, low BMI, presence of rhinitis	
Omalizumab and Mepolizumab	Thomas et al. (2023) [129]	Better lung function	12 months
		Lower body mass index	
		Mild disease	
		Absence of comorbidities such as obesity, depression and osteoporosis	
Mepolizumab and Benralizumab	Carpagnano et al. (2024) [70]	Higher baseline BEC	12 months
		Better lung function	
		Comorbid CRwNP	
		Frequent administration of OCS or inhaled reliever therapy (as needed SABA or ICS‐Formoterol)	
Omalizumab, Mepolizumab, Reslizumab, Benralizumab	Valverde‐Monge et al. (2024) [130]	Higher FEV1%	12 months
		Higher ACT score	
		N‐ERD before treatment	
Omalizumab, Benralizumab, Mepolizumab, Reslizumab, Dupilumab	Hansen et al. (2024) [71]	Lower BMI	12 months
		Shorter duration of disease	
Mepolizumab, Benralizumab, Reslizumab	Bal et al. (2025) [73]	Absence of maintenance OCS use	12 months
		Higher ACT score	
		FEV1 in liter	
Mepolizumab, Benralizumab, Reslizumab	Blouin et al. (2025) [74]	bBEC ≥ 300	12 months

Abbreviations: ACQ, asthma control questionnaire; ACT, Asthma control test; bBEC, baseline blood eosinophil count; BMI, Body mass index; CRwNP, Chronic rhinosinusitis with nasal polyps; FeNO, fraction exhaled nitric oxide; FEV, forced expiratory volume 1; ICS, inhaler corticosteroid; N‐ERD, Nonsteroidal antiinflammatory drug‐exacerbated respiratory disease; OCS, oral corticosteroids; SABA, short acting beta2 agonist.

### 8.1 Anti IgE Monoclonal Antibody (Omalizumab)

Omalizumab is a well‐known biologic that inhibits the binding of free serum and tissue IgE to FceRI/FceRII receptors expressed on basophils, mast cells, and other immune cells [109]. Randomized controlled trials and real‐world studies confirm the efficacy of Omalizumab in the treatment of moderate to severe allergic asthma. Regarding remission, a recent real‐world study evaluated clinical remission in patients receiving omalizumab and classified it as patients receiving no oral corticosteroid (OCS) therapy; no exacerbations; ACQ ≤ 1, ACT ≥ 20, or both; and FEV1 of ≥ 80% predicted [110]. There are real‐life studies evaluating clinical remission with a large number of biologics, including Omalizumab, but clinical remission criteria are heterogeneous and remission rates vary accordingly [14, 42, 68, 69, 71, 72, 75, 76, 128, 129, 130]. Sposato et al. reported that clinical remission in omalizumab‐treated patients was associated with younger age, lower body mass index, earlier onset of asthma, absence of sinusitis or nasal polyposis, no history of hypertension or chronic heart disease, and fewer exacerbations [68].

### 8.2 Anti IL‐4Ra Monoclonal Antibody (Dupilumab)

Dupilumab reduces IL‐4 and IL‐13 cytokine‐induced responses, including the production of pro‐inflammatory type 2‐cytokines and immunoglobulin E, by specifically interacting with IL‐4Ra [111]. A post hoc analysis of the LIBERTY ASTHMA QUEST and TRAVERSE trials was conducted to evaluate the efficacy of Dupilumab in patients with uncontrolled, moderate‐to‐severe asthma. The analysis used a multicomponent endpoint that included various dimensions of clinical asthma remission. The parameters included the absence of exacerbations and OCS, ACQ‐5 < 1.5, and post‐bronchodilator FEV1 ≥ 80% at 6 and 12 months [112]. The post hoc analysis of the QUEST and TRAVERSE trials for long‐term clinical remission used different lung function criteria: a decline in pre‐ or post‐bronchodilator FEV1 of less than 5% from the parent study baseline [41]. In addition to the analysis of the above studies, there are also real‐world studies with Dupilumab that have encouraging remission rates [14, 58, 59, 60, 68, 69, 71, 72, 75, 76, 113, 114, 115, 116, 131]. Between 18.1% and 49.4% of patients achieved remission in the clinical remission trials evaluating Dupilumab alone [41, 58, 59, 60, 111, 112, 114, 115, 116]. Higher baseline blood eosinophil counts (> 300/μL), male sex, aspirin‐exacerbated respiratory disease, and concomitant CRSwNP were identified as predictors of remission in patients treated with dupilumab [60, 114].

### 8.3 Anti‐IL‐5 Monoclonal Antibodies (Mepolizumab, Reslizumab)

IL‐5 is the key cytokine that regulates the development, differentiation, recruitment, activation, and survival of eosinophils. Mepolizumab and Reslizumab are both IL‐5‐targeting biologics [48]. In terms of clinical remission, encouraging results can be found in literature, and the majority of data were available for Mepolizumab. The recent post hoc analysis, REDES study, used two definitions of clinical remission with Mepolizumab treatment. To define clinical remission at 12 months, four criteria were used: OCS‐free, exacerbation‐free, ACT ≥ 20, and post‐BD FEV1 ≥ 80%. The second one was a three‐component clinical remission definition that included meeting the following criteria: OCS‐free, exacerbation‐free (for 52 weeks); and ACT score ≥ 20 [61]. Bagnasco et al. evaluated clinical remission using different pulmonary function criteria, which included improvement or a maximum decline from a previous test of 150 mL [62]. Post hoc analyses showed that remission rates for Mepolizumab ranged from 27.4% to 30% over a period of 12 months [61, 122]. Real‐life studies have evaluated clinical remission with a large number of biologics, including Mepolizumab and Reslizumab, but the criteria for clinical remission are heterogeneous, and remission rates vary accordingly [14, 42, 67, 68, 69, 70, 71, 72, 73, 74, 75, 76, 82, 119, 120, 121, 122, 123, 124, 128, 129, 130, 131]. For mepolizumab, higher baseline lung function (FEV_1_ and FEV_1_ % predicted), higher blood eosinophil counts, lower oral corticosteroid requirements, fewer exacerbations, improved asthma control (ACQ‐5 at 3 and 6 months), higher Asthma Quality of Life Questionnaire scores, lower FeNO levels, and the presence of nasal polyps were identified as predictors of remission [62, 68, 120].

### 8.4 Anti‐IL‐5R Monoclonal Antibody (Benralizumab)

Benralizumab is an interleukin‐5 receptor alpha‐targeting monoclonal antibody that results in rapid and almost complete depletion of eosinophils [48]. Previous pivotal phase 3 studies (SIROCCO, CALIMA, and ZONDA), real life studies showed effectiveness and safety of Benralizumab. A post hoc analysis of the SIROCCO, CALIMA and ZONDA trials was conducted to evaluate the efficacy of Benralizumab in patients with uncontrolled, moderate‐to‐severe asthma. The analysis used a multicomponent endpoint that included various dimensions of clinical asthma remission. The parameters included the absence of exacerbations and OCS, ACQ‐6 < 1.5 or ACT ≤ 0.75, and Pre‐BD FEV1 increase ≥ 100 mL at 6 and 12 months [63]. There were differences in terms of clinical remission criteria in post hoc analysis and retrospective studies [14, 42, 63, 64, 65, 68, 69, 70, 71, 72, 73, 74, 75, 76, 82, 117, 118, 125, 126, 127, 130, 131]. Between 14.5% and 69.5% of patients achieved remission in the clinical remission trials evaluating Benralizumab alone [63, 118, 125, 127]. For benralizumab, predictors of remission included low body mass index, the presence of rhinitis, nasal polyposis, osteoporosis, oral corticosteroid dependency, predicted pre‐bronchodilator FEV_1_ ≥ 80% at baseline, and predicted baseline FEF25%–75% < 65% [68, 127].

### 8.5 Anti‐TSLP Monoclonal Antibody (Tezepelumab)

Tezepelumab is a monoclonal antibody that targets TSLP and reduce the severity of eosinophilic inflammation, airway hyperresponsiveness and asthma exacerbations in asthmatic patients [1, 39, 48]. In terms of clinical remission, few evidences can be found with Tezepelumab. In the post hoc analysis of NAVIGATOR trial, remission criteria included ACQ‐6 ≤ 1.5, FEV1 > 95% of baseline at the end of each year, no OCS use and exacerbation. They showed that 28.5% of patients achieved remission at the end of 12 months [43]. There are two retrospective studies on asthma remission with Tezepelumab [44, 66]. Sumi et al.'s studies differ from other Tezepelumab studies in that their patients had an ACT score of at least 23 [66]. This could explain the lower remission rate compared to other studies [43, 44, 66]. Among patients receiving tezepelumab, female sex, lower body mass index, adult‐onset asthma, higher FeNO, and lower ACQ‐6 scores emerged as significant predictors of remission [44].

## 9 Future Perspectives

As proposed by Pavord et al. [61] the concept of remission in asthma is evolving from the assessment of clinical remission while on treatment toward the more challenging goal of clinical remission off‐treatment. This shift highlights the need for a deeper understanding of disease mechanisms and for reliable tools capable of identifying patients who may achieve sustained disease control without ongoing therapy.

New therapeutic options for asthma are under investigation for their efficacy and potential impact on remission. These include novel agents such as Rilzabrutinib [133], an oral Bruton's tyrosine kinase inhibitor. In T2‐low asthma, ongoing studies are evaluating monoclonal antibodies targeting IL‐33, such as Itepekimab, as well as approaches involving ST2 receptor inhibition and antagonists of IL‐6, IL‐17, and the chemoattractant receptor‐homologous molecule expressed on Th2 cells (CRTH2) [134, 135].

Nowadays and in the future, the development of novel serum or sputum biomarkers may improve asthma endotype characterization and patient stratification, including the development of epithelial‐focused therapeutic strategies. Given the central role of epithelial barrier alterations in airway remodeling, future targets could interfere with the pathways of TGF‐β, platelet‐derived growth factor (PDGF), fibroblast growth factor (FGF), epidermal growth factor (EGF), vascular endothelial growth factors (VEGFs), and chemokines such as CXCL2, CXCL3, and IL‐8/CXCL8 [107].

Two emerging biomarkers are Ezrin and CHI3L1. Ezrin is a membrane‐associated cytoskeleton protein involved in cell morphology, intercellular adhesion, and epithelial barrier integrity; its downregulation can be a marker for monitoring asthma severity, since it negatively correlates with serum periostin and IL‐13 levels. CHI3L1 (Chitinase‐3‐like‐protein 1), also known as YKL40, correlates with non‐T2 inflammatory mediators such as IL‐1B and IL‐6 and induces mesenchymal transition and subepithelial fibrosis. Its expression is increased in severe asthma and airway remodeling. In addition, protocadherin‐1 (PCDH1), a susceptibility gene for airway hyperresponsiveness, represents a potential biomarker applicable to both pediatric and adult populations [106].

Current evidence on asthma remission largely derives from retrospective, post hoc, or registry‐based analyses, underscoring the need for prospective studies specifically designed to assess remission using standardized and multidimensional outcome measures. In this context, earlier intervention in the disease course may be associated with a higher likelihood of remission, potentially preceding the development of irreversible airway remodeling and lung function decline driven by prolonged type 2 (T2) inflammation; however, evidence supporting the early use of biologics remains limited and requires further investigation, particularly in patients with clear markers of active T2 inflammation.

Another important and increasingly debated aspect of asthma remission is its long‐term sustainability, as accumulating real‐world evidence suggests that remission is often a dynamic and potentially reversible state, with a subset of patients cycling in and out of remission due to early or late loss of response to biologic therapies, underscoring the need for longitudinal assessment and deeper mechanistic insights into the durability of treatment response. This dynamic pattern, highlighted in recent work [136], underscores that remission is not always permanent and may fluctuate with changes in inflammatory activity, comorbidities, adherence, or external triggers. The forthcoming reports from international registries are expected to clarify the biological and clinical determinants of remission durability. Integrating the concept of *remission sustainability* into long‐term follow‐up protocols could refine treatment objectives and enhance personalized management strategies for patients receiving biologic therapy.

## 10 Conclusions

Clinical remission with treatment has recently become an achievable goal in severe asthma. The success of biologics in achieving remission in severe asthma is encouraging. Current evidence suggests that younger age at asthma onset, milder symptoms at diagnosis, male sex, and higher baseline lung function appear to be positive predictors. However, the use of variable criteria in these studies does not allow complete standardization of results. As a future perspective, additional outcomes may be considered in the definition of remission, such as the reduction of inhaled corticosteroids and the improvement of bronchial hyperresponsiveness. More research is needed to standardize the criteria and to understand which asthma management strategy may increase the likelihood of achieving remission.

## Author Contributions

Conceptualization and methodology: Fatma Esra Gunaydin, Bianca Olivieri, and Marco Caminati. Investigation and formal analysis: Fatma Esra Gunaydin, Giulia Marcassa, and Bianca Olivieri. Writing – original draft and writing – review and editing: Fatma Esra Gunaydin, Giulia Marcassa, Bianca Olivieri, Gianenrico Senna, and Marco Caminati. Supervision: Gianenrico Senna and Marco Caminati.

## Funding

The authors have nothing to report.

## Conflicts of Interest

The authors declare no conflicts of interest.

## Data Availability

The data that support the findings of this study are available from the corresponding author upon reasonable request.
